# PCSK9 in Liver Cancers at the Crossroads between Lipid Metabolism and Immunity

**DOI:** 10.3390/cells11244132

**Published:** 2022-12-19

**Authors:** Malak Alannan, Nabil G. Seidah, Aksam J. Merched

**Affiliations:** 1 Bordeaux Institute of Oncology (BRIC), INSERM U1312, University of Bordeaux, F-33000 Bordeaux, France; 2 Laboratory of Biochemical Neuroendocrinology, Montreal Clinical Research Institute, IRCM, University of Montreal, Montreal, QC H2W 1R7, Canada

**Keywords:** PCSK9, metabolism, liver cancer, immuno-oncology

## Abstract

Metabolic rewiring and defective immune responses are considered to be the main driving forces sustaining cell growth and oncogenesis in many cancers. The atypical enzyme, proprotein convertase subtilisin/kexin type 9 (PCSK9), is produced by the liver in large amounts and plays a major role in lipid metabolism via the control of the low density lipoprotein receptor (LDLR) and other cell surface receptors. In this context, many clinical studies have clearly demonstrated the high efficacy of PCSK9 inhibitors in treating hyperlipidemia and cardiovascular diseases. Recent data implicated PCSK9 in the degradation of major histocompatibility complex I (MHC-I) receptors and the immune system as well as in other physiological activities. This review highlights the complex crosstalk between PCSK9, lipid metabolism and immunosuppression and underlines the latest advances in understanding the involvement of this convertase in other critical functions. We present a comprehensive assessment of the different strategies targeting PCSK9 and show how these approaches could be extended to future therapeutic options to treat cancers with a main focus on the liver.

## 1. Introduction to Liver Cancers

### 1.1. The Liver, a Multifunctional Organ

Being the largest and most functionally diverse organ in the human body, the liver is a heterogeneous organ composed of several cell types, including hepatocytes (primary epithelial cell population), cholangiocytes (also known as biliary epithelial cells), stellate cells, Kupffer cells and liver sinusoidal endothelial cells. Considering their unique functions, these cell types work together to regulate the normal liver function at different levels. Thus, the liver is known to be a critical hub for multiple physiological processes, which include the metabolism of nutrients (glucose, lipids and amino acids), the regulation of blood volume, lipid and cholesterol homeostasis, the support of the immune system and the metabolism of xenobiotic drugs. The energy provided by the processing and metabolism of nutrients is the driving fuel for all of these processes. The liver is a highly adaptive organ which has the capacity to store glucose in the form of glycogen under feeding conditions and can breakdown glycogen via glycogenolysis or assemble glucose through gluconeogenesis under fasting conditions. It can also oxidize lipids for energy when the glycogen reservoir is depleted, while it regulates the storage of excess lipids in adipose tissues. Moreover, it is the main site for protein and amino acid metabolism where it can process the latter for energy production, produce the majority of proteins secreted in the blood and can eliminate nitrogenous wastes resulting from protein degradation via urea metabolism [1,2].

The liver is highly vascularized in its nature, making it the most common site for cancer development, especially metastatic cancers. According to the GLOBOCAN 2020 estimates of cancer incidence and mortality, liver cancer ranks the sixth among the most commonly diagnosed cancers and the third leading cause of cancer-related deaths worldwide. Every year more than 900,000 new cases of liver cancers are diagnosed and 830,000 deaths are recorded. Notably, the incidence and mortality rates are 2 to 3 times higher in males than in females [3].

### 1.2. Adult Liver Cancer: Hepatocellular Carcinoma (HCC)

Hepatocellular carcinoma (HCC) is the most common form of adult primary liver cancer, accounting for ~90% of all cases [4]. It is associated with poor prognosis and an overall survival rate of 3–5% [5]. It is of no surprise that the incidence rate of HCC is increasing worldwide as it is accompanied by many different risk factors, including chronic hepatitis B and C viral (HBV/HCV) infection, alcohol abuse, exposure to aflatoxin B1 and all cirrhosis-causing conditions, such as non-alcoholic fatty liver diseases (NAFLD) [6]. For instance, non-alcoholic steatohepatitis (NASH) (a form of NAFLD) associated with metabolic syndrome or diabetes mellitus is considered the second most common etiology of HCC [7]. Nonetheless, the etiology of HCC can also arise from alterations in oncogenes and tumor-suppressor genes (e.g., p53), genes leading to aberrant signaling pathways (e.g., Wnt-β-catenin pathway), the overexpression of epidermal growth factor (ErbB) receptors and telomerase activation (*TERT* mutations), as well as chromosomal instabilities [6,8].

The management of HCC not only includes its early diagnosis but also the staging of the disease and the suitable treatments of patients and those who are at risk of developing the disease, which is why the surveillance of HCC is very important, where subjects at risk are periodically diagnosed for HCC development. Cancer surveillance allows the early detection of tumors in patients at high risk, thus increasing the opportunity for curative treatments and improving survival by decreasing the disease-related mortality. The American Association for the Study of Liver Diseases (AASLD) and European Association for the Study of the Liver (EASL) guidelines suggest surveillance programs of high-risk patients, including all cirrhotic patients (HBV/HCV infection, AFLD, NAFLD/NASH), HBV carriers from regions of high HBV incidence, such as Asia and Africa, as well as HCV patients for whom infection is not associated with cirrhosis [8,9]. The diagnosis of HCC can be carried out using the following: serologic testing (α-fetoprotein (AFP), glypican 3 (GPC3)), diagnostic imaging (multidetector computed tomography (MDCT) or magnetic resonance imaging (MRI)) and histology [9,10,11,12].

Once diagnosed, patients will be stratified according to the Barcelona Clinic Liver Cancer (BCLC) staging system which relies on the number and size of the tumors as well as the liver function and health status of the patient (Eastern Cooperative Oncology Group [ECOG] Performance Status [PS]) [4,8]. So far, BCLC is the best staging system proposed, as it is the most commonly used system for HCC. The algorithm used by this system links the tumor stages (BCLC 0, A, B, C and D) to their corresponding treatments [13]. Treatments are the fundamental therapies that aim to either cure HCC using radical therapies or find palliative options that help to improve overall survival. Radical therapies include ablation, resection and transplantation, whilst palliative therapies involve chemoembolization and systemic therapies such as: multikinase inhibitors (sorafenib), immune-checkpoint inhibitors (atezolizumab: anti-programmed cell death ligand 1 (PDL1) antibody) alone or in combination with bevacizumab (anti-vascular endothelial growth factor (VEGF) antibody) which was recently approved by the Food and Drug Administration (FDA) on May 2020 [4,13,14].

### 1.3. The Pediatric Liver Cancer: Hepatoblastoma (HB)

Hepatoblastoma (HB) is a rare malignant embryonic tumor accounting for only 1% of all pediatric neoplasms, yet it is the most common primary hepatic tumor in babies [15]. Approximately one in million children are affected by HB, which usually develops between six months and three years after birth in around 95% of cases, with a median age of 18 months [16]. The remaining 5% are diagnosed in children over 4 years old and exceptionally in some adult cases [16,17]. The etiology of HB is still not fully understood; however, tumorigenesis is believed to arise from immature hepatocytes that differentiate into other cell types, such as hepatocytes, epithelial, biliary and mesenchymal cells. This explains the heterogeneity observed at the cellular and histological levels among patients—56% epithelial or 44% mixed forms (epithelial and mesenchymal), with the former being associated with poor prognosis [15].

Most HB tumors develop sporadically; however, one-third of the cases could be associated with inherited syndromes, such as familial adenomatous polyposis (FAP) and Beckwith–Wiedemann syndrome (BWS), and gestational factors, such as pre-mature birth and very low birth weight, as well as some environmental factors, such as maternal exposure to alcohol and smoking [18]. The most common genetic mutation detected in HB involves the Wnt signaling pathway (β-catenin (*CTNNB1*), adenomatous polyposis coli (*APC*) and axis inhibition protein 1 (*AXIN1*)), reflecting its importance in the tumorigenesis of HB [19,20,21,22]. The overall survival rate of HB recorded so far is around 80% [23], thanks to the modern imaging techniques used to detect it [24] as well as the adapted chemotherapeutic regimes being used [25].

The diagnosis of HB is carried out in a similar manner to that of HCC using imaging modalities, serologic testing or histology. This disease is characterized by elevated levels of AFP, which is not only helpful for diagnosis but also in assessing the efficacy of a given treatment [26]. Regarding which, treatments include surgical resection, if possible, chemotherapy (Cisplatin) or liver transplantation. Thanks to the combined efforts of four international groups, a standardization of risk criteria and patient stratification has been put in place to help treat HB patients [27].

Despite all the advances made in stratifying HB patients (and even HCC patients) before choosing the suitable treatment options and hence improving their lifestyle, it is hard to neglect the fact that some patients do not respond to such treatments, with some developing resistance to chemotherapy or even relapsing in a short period of time. Because of this, it is more urgent than ever to find alternative therapeutic approaches that are less toxic and more efficient by conducting in-depth research on the molecular biology of HB and HCC. Recently, two emerging hallmarks of cancer, i.e., energetic metabolism reprogramming and the escaping of immune destruction, have been the focus by many researchers who aim to understand the possible connections between both, thus attaining new therapeutic strategies.

## 2. Hallmarks of Cancer: Deregulated Metabolism and Inflammation

It took centuries for scientists to solve the mysterious way by which cancer develops. Historically, it was believed that cancer was contagious and could spread through infections. It was not until the 20th century that the real problem began to be solved. In year 2000, the six hallmarks of cancer were introduced by Hanahan and Weinberg [28], which included the ability of tumor cells to be self-sufficient from the surrounding growth signals along with being resistant to any anti-growth ones, having uncontrolled proliferation, escaping apoptosis and inducing angiogenesis, as well as being capable of invasion and metastasis. These hallmarks were then defined as acquired novel capabilities necessary for tumor growth and proliferation.

Eleven years later, Hanahan and Weinberg published a new update to these hallmarks of cancer, which they termed “the next generation”, and among which two were novel: energetic metabolism reprogramming and escaping immune destruction [29]. A growing body of research suggests that these two hallmarks are implicated in the pathogenesis of most, if not all, cancers. The deregulated metabolism of liver cancer cells, specifically their lipid metabolism and relation to inflammation, has been discussed and reviewed in Alannan et al. [30].

It is important to note that the tumor microenvironment (TME) is a unique niche consisting of complicated components, such as metabolites and immune cells, which play a vital role in tumorigenesis. The reciprocation between metabolism and immunology known as immunometabolism has been under the spotlight recently. This is due to the recent discoveries that revealed the major impact of cellular metabolism on the behavior of cells [31]. For instance, in liver cancer, the TME is characterized by the presence therein of many immune cells, such as macrophages, T cells, B cells, dendritic cells, natural killer (NK) cells, etc. These cells are capable of modulating their own metabolism in order to create an immunosuppressive environment for cancer cells, therefore favoring tumor survival and progression [32]. This complex crosstalk between cancer cells and metabolically reprogrammed immune cells needs further exploration, especially at the molecular level. However, targeting immunometabolism is a promising therapeutic approach that is still in its infancy.

## 3. PCSK9, a Key Proprotein Convertase

### 3.1. Background

Amongst the 30,000 genes encoded by the mammalian genome, ~1.7% are proteolytic enzymes that belong to one of six classes of proteases: serine, threonine, metallo, cysteinyl, glutamyl and aspartyl [33]. Generally, proteases are capable of irreversibly cleaving a wide number of substrates that include hormones, growth factors, receptors, other proteases and cytokines. This cleavage can result in either the activation or inhibition of the target molecule.

In 2003, Seidah et al. [34] identified a putative proprotein convertase-named neural apoptosis-regulated convertase 1 (NARC-1) through the cloning of complementary deoxyribonucleic acids (cDNAs) that were upregulated in primary cerebellar neurons after inducing apoptosis by serum deprivation. However, at that time, little was known about the activity, cleavage specificity, cellular/tissue expression and function of this protein. All that was known was its gene localization in the human chromosome 1p33-p34.3 and the higher expression of NARC-1 messenger ribonucleic acid (mRNA) in the liver than in the gut, testis and kidney. Through further investigation, Seidah and his group identified NARC-1 as a soluble mammalian protein convertase member of the proteinase K subfamily of subtilases that is composed of multiple domains: signal peptide, pro-domain, catalytic domain, a hinge region and, finally, a C-terminal Cys-His rich domain (CHRD) [34]. The proPCSK9 zymogen undergoes an autocatalytic prosegment cleavage in the endoplasmic reticulum (ER), resulting in a mature prodomain- PCSK9 complex which can then exit the ER and be secreted. It is important to note that the cleaved prosegment of NARC-1 remains associated with it even after being secreted. Moreover, a very high expression of NARC-1 was recorded in the neuroepithelioma, hepatic and colon carcinoma cell lines. This expression seemed to be related to cells that can proliferate and differentiate, such as hepatocytes [34].

The discovery of NARC-1 by Seidah was the basis for revealing that gain-of-function (GOF) mutations in this gene, leading to higher production, were the cause of familial hypercholesterolemia (i.e., a genetic form of extremely high levels of low-density lipoprotein cholesterol (LDL-C) [35], a critical risk factor for coronary heart disease). *Nature Genetics* editors suggested changing the NARC-1 nomenclature to PCSK9, as they considered this name as being more adapted to standard nomenclature. PCSK9 stands for proprotein convertase subtilisin/kexin type 9, and it is a serine protease that belongs to the family of proprotein convertases (PCs). This family comprises eight other members, namely PC1, PC2, Furin, PC4, PC5, PACE4, PC7 and subtilisin-kexin isozyme 1 (SKI-1/S1P). All of these proteins function as proteases that can cleave many substrates, including precursor proteins, growth factors, receptors, hormones and even transmembrane proteins, such as membrane-bound transcription factors [36]. However, PCSK9 is an exception compared to the other PC family members. This protein exerts its action only through self-activation, making it the one and only substrate for itself [37]. As mentioned above, the autocatalytically cleaved inhibitory prosegment of PCSK9 remains associated with mature PCSK9 even after secretion making it the only PC that is secreted as a catalytically inactive enzyme [34,38]. The history of the discovery of PCSK9 and that of the eight other PC family members has been recently reviewed by Seidah [39].

### 3.2. Structure

**Figure 1:**
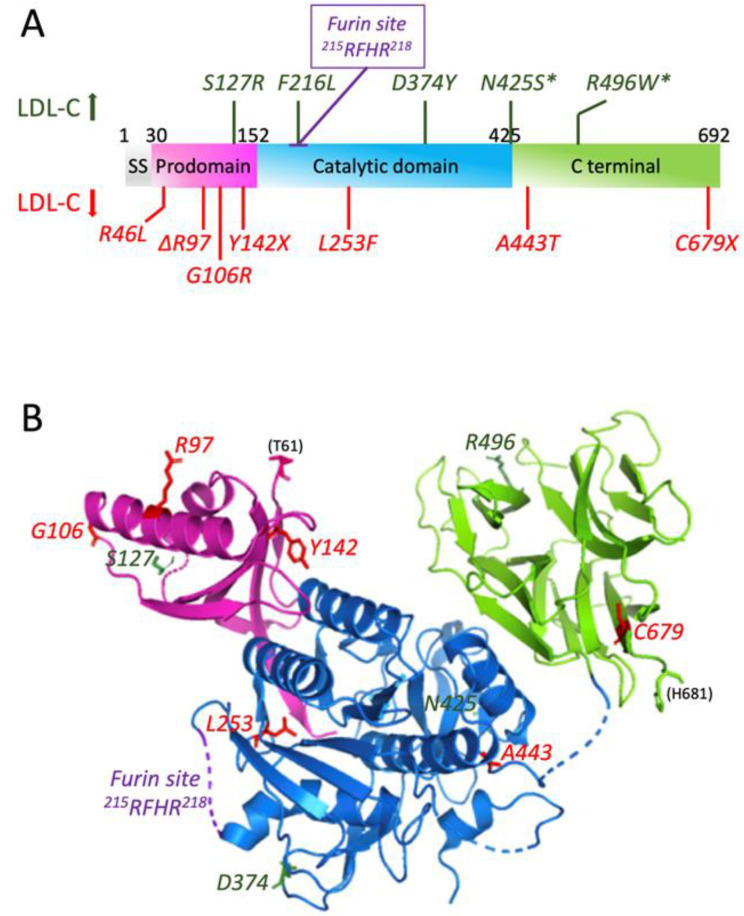
PCSK9 domains, mutations and 3D structure. (**A**) Different domains of PCSK9 along with the naturally occurring mutations (adapted with permission from [47], 2022, Elsevier). The mutations increasing LDL-C are in green; those decreasing LDL-C are in red. (**B**) 3D structure representation of PCSK9 from amino acid 61 to 681 (PDB 2PMW) generated with Pymol 2.4.2.

The human *PCSK9* gene encodes a 74.3-kDa zymogen composed of 692 amino acids. This protein has multiple domains, as first revealed by X-ray crystallography [40,41] and reviewed by Seidah and Prat [36] and Lambert et al. [42]: signal peptide (aa 1-30), prodomain (aa 31–152), catalytic domain (aa 153–451) and C-terminal CHRD domain (aa 452–692), which is rich in cysteine and histidine (Figure 1). The only domain lacking in this protein as compared to its family members is the P-domain [43], which is known to be involved in protein folding and the regulation of the protease activity [44]. Unlike its respective mates in the family, which primarily act at proteases to cleave after basic residues (PC1 to PC7) [45] or following non-basic residues (SKI-1/S1P) [46] in the presence of calcium, PCSK9 undergoes an autocatalytic intramolecular cleavage after a non-basic Gln_152_↓ residue, without any requirement for calcium [38,47] in order to form a ~14-kDa prodomain that remains non-covalently tightly associated with a ~60-kDa domain (catalytic and C-terminal domain) in the secretory pathway [34,38,40,41,42]. The self-inhibition of the prodomain potently blocks the activity of the catalytic domain. However, complex prodomain-PCSK9 exhibits a high affinity binding towards the epidermal growth factor-like repeat A (EGF-A) domain of the LDL receptor (LDLR), resulting in an efficient degradation of LDLR [37,48,49] in endosomes/lysosomes [50]. Moreover, the PCSK9 prodomain and its mature form both undergo a number of post-translational modifications, such as Tyr-sulphation [38,51], Asn-glycosylation [34] and Ser-phosphorylation [52], some of which result in PCSK9 GOF on the LDLR.

On another note, multiple mutations of PCSK9 can be found, which either induce higher activity, associated with hypercholesterolemia, or lower activity, associated with hypocholesterolemia (Figure 1). These mutations will be discussed in the next section.

### 3.3. Function

The main role of PCSK9 is to promote the degradation of the hepatic LDLR present on the surface of hepatocytes, thereby blocking the primary pathway of LDL-C uptake from the circulation and leading to an increase in blood LDL-C levels. Lagace et al. [49] were the first to show the molecular mechanism by which PCSK9 represses LDLR. PCSK9 is secreted from cells and is subsequently internalized with LDLR to promote its degradation. This internalization process is dependent on the presence of an adaptor protein called autosomal recessive hypercholesterolemia (ARH) that binds to the cytosolic tail of LDLR [49,53]. However, another study has shown that PCSK9 can still degrade LDLR even in ARH knockout mice [54], and this is possibly achieved either en route from the synthesis of LDLR in the ER to the cell surface (intracellular pathway) or on the cell surface upon the internalization of the PCSK9-LDLR complex in clathrin-coated vesicles [55]. The proposed intracellular pathway as an alternative mode of action of PCSK9 is supported by the presence of two naturally occurring mutants—S127R and D129G—whose secretion is impaired, yet their function is intact [56].

**Figure 2:**
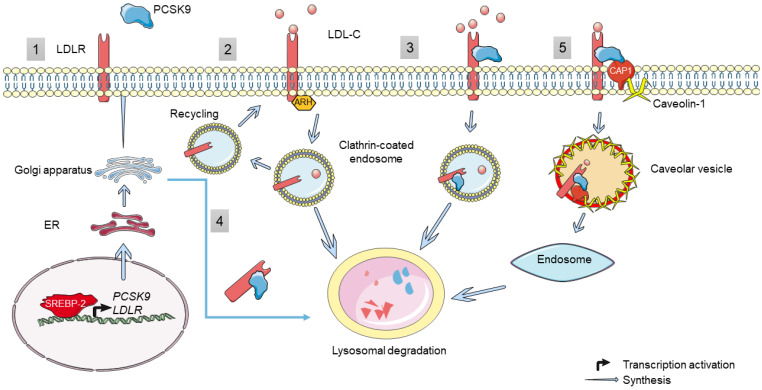
PCSK9, genetic regulation and function. (1) The synthesis of PCSK9 and LDLR is regulated transcriptionally by SREBP-2. Once translated, they travel through the ER and Golgi apparatus for maturation before being secreted. (2) LDLR expressed on the cell surface binds to LDL-C and is internalized in an ARH-dependent manner via clathrin-coated endosomes. Inside the endosomes, LDL-C dissociates from the receptor and is directed toward lysosomal degradation, whilst LDLR is recycled back to the cell surface. (3) The extracellular and (4) intracellular pathway regulation of LDLR by PCSK9: in both pathways, PCSK9 binds to the EGF-like domain of LDLR and targets it for lysosomal degradation instead of recycling. (5) A novel extracellular pathway has been proposed by which PCSK9 induces LDLR degradation by interacting with CAP-1, which in turn binds to caveolin-1 and induces caveolin-dependent endocytosis followed by the lysosomal degradation of the LDLR/PCSK9/CAP1 complex. However, its validation is not confirmed.

Thus, we can distinguish two pathways by which PCSK9 regulates LDLR: intracellular and extracellular pathways (Figure 2). Active PCSK9 binds through its catalytic subunit to the LDLR on its EGF-A domain. In the intracellular pathway, this binding will facilitate the transport of LDLR from the trans-Golgi network to lysosomes, instead of going to the cell surface, with the help of clathrin light chains, thus mediating its degradation [55]. This is confirmed by using brefeldin A—a fungal toxin that induces that dissociation of the Golgi complex. Brefeldin A prevented LDLR degradation by PCSK9, thus suggesting that PCSK9 can cause LDLR degradation as it migrates from ER to the cell membrane [57]. On the contrary, in the extracellular pathway, mature PCSK9 is released from the Golgi apparatus and binds to the LDLR expressed on the cell surface, and together, they are internalized in heavy chain clathrin-coated endosomes, which later fuse with lysosomes to enter into the degradation process [55]. It is important to note that this internalization process necessitates the presence of the ARH adaptor protein [37,58]. Moreover, a third pathway by which PCSK9 induces LDLR degradation was recently discovered to be mediated by caveolin in a cyclase-associated protein 1 (CAP1)-dependent manner [59]. Through this pathway, CAP1 is capable of binding to the C-terminal cysteine-rich domain (CRD) of PCSK9 as well as to caveolin-1 through two different domains, the Src homology 3 binding domain (SH3BD) and adenylyl cyclase-binding domain (ACBD), respectively. This interaction guides the PCSK9/LDLR complex towards caveolae-dependent endocytosis and further lysosomal degradation [59]. However, recent studies in Seidah’s group revealed that the cytosolic CAP1 is actually secreted and binds the CHRD extracellularly, thereby facilitating the sorting of LDLR/PCSK9/CAP1 complex from clathrin-coated endosomes towards the lysosomes for degradation (in preparation).

The secreted mature form of PCSK9 (~60 kDa) can be cleaved by the action of two convertases of the same family (furin and PC5/6A) after the recognition sequence R215-F216-H217-R218, giving rise to a truncated PCSK9 protein (50 kDa) and resulting in an effective loss-of-function (LOF) [51]. In humans, up to 40% of the circulating PCSK9 are found in this truncated inactive form [60,61].

As mentioned earlier, PCSK9 naturally occurring mutations can be present, rendering the protein either more or less active. This results in GOF or LOF variants of PCSK9, respectively. These mutations and their impact have been studied in diseases such as Familial Hypercholesterolemia (FH), hypocholesterolemia and coronary heart disease (CHD).

#### 3.3.1. GOF Mutations of PCSK9

Many missense mutations exist in *PCSK9* gene, which can cause dominant hypercholesterolemia in patients, either by increasing the activity of PCSK9 or by giving it a new activity. Three of these mutations, S127R, F216L and D374Y, have been detected in patients with symptoms of FH and FDB. Other mutations have been detected only in patients who also have mutations in *LDLR* gene, i.e., N425S and R496W (Figure 1) [47]. In these hypercholesterolemia patients, the plasma levels of LDL-C were around 50% higher than in those with either mutation alone [62].

Both S127R and F216L mutant forms of PCSK9 have been tested in vivo in comparison to the wild type (WT) PCSK9. All of these forms were highly expressed in mice livers, and they were capable of remarkably decreasing the protein levels of LDLR without any change at the mRNA levels [38,54,63]. On the contrary, in vitro experiments showed variable effects of PCSK9 on LDLRs. In some cells, such as human hepatoma cells (Huh7 and HepG2) or human embryonic kidney cells (HEK293), PCSK9 significantly decreases the levels of LDLRs. However, this was not the case in other cells, such as fibroblasts or Chinese hamster ovarian (CHO-K1) cells. This difference in terms of action between cell lines can be attributed to some missing factor essential for PCSK9 function [47].

In addition, the D374Y mutant of PCSK9 [64] has been shown to increase the affinity of PCSK9 to the EGF-A domain of LDLR by 25-fold compared to WT PCSK9 and is 10-fold more active than the WT in decreasing the levels of LDLR [40].

#### 3.3.2. LOF Mutations of PCSK9

Nonsense, missense and even in-frame deletion mutations have been found in the *PCSK9* gene to cause hypocholesterolemia by the increased clearance of LDL-C (Figure 1). Three LOF mutations in African Americans (Y142X and C679X) and Caucasians (R46L) have been shown to afford a great protection against CHD. A 15-year prospective study performed in these populations has shown that nonsense heterozygote mutations of *PCSK9* not only reduced LDL-C levels by 28% but also decreased the frequency of CHD by 88%. In case of R46L allele mutation, the frequency of CHD was decreased by 50% as the average reduction of LDL-C levels was only 15% [65]. These observations indicate that LOF mutations in *PCSK9* reduce the risk of CHD better than using statins in a short-term clinical trial [66]. In addition, Y142X mutation results in undetectable protein expression, whilst the C679X mutant is not secreted into the medium of human liver cells because of the misfolding and retaining in the ER [38]. L253F mutation is known to impair the autocatalytic cleavage and thus the secretion of PCSK9. Mutations in the prodomain (Δ97, G106R) and catalytic domain (L253F) also interfere with the autocatalytic cleavage of the protein [40,47]. Finally, the A443T mutant form was shown to be O-glycosylated and hence more susceptible to furin cleavage, leading to LOF [51].

Interestingly, a novel LOF mutation, Q152H, was discovered in a white French-Canadian family that results in very low plasma PCSK9 concentration and, subsequently, a lower concentration of circulating LDL-C. The amino acid substitution at position 152 results in the impairment of autocatalytic cleavage of proPCSK9, hence limiting its processing and secretion from the ER [67,68]. Not only is this variant capable of protecting against cardiovascular diseases, but it can also protect against liver injuries caused by ER stress. This was very recently demonstrated by Lebeau et al. [69] who identified a cochaperone function for the Q152H variant retained in ER, which increased the abundance of ER chaperones and glucose-regulated proteins of 78 and 94 kDa (GRP78 and GRP94), resulting in protection against ER stress-induced liver injury.

The main effect exerted by PCSK9 is on the expression of LDLR in hepatocytes, thus regulating directly LDL-C concentrations in plasma and indirectly that of oxidized LDL (oxLDL) [70]. However, the PCSK9 regulatory mechanism can have consequences on extra-hepatic tissues in which it is expressed, such as kidney and heart, which usually respond to LDL-C and oxLDL. In such tissues, the cholesterol concentration is dependent on secretion rather than uptake by LDLR, LDLR-related protein 1 (LRP-1=CD91) and lectin-like oxidized LDL receptor 1 (LOX-1). The endogenous expression level of these receptors is controlled by PCSK9 along with their ligands [71]. Moreover, PCSK9 can target other members of LDLRs, such as very low-density lipoprotein receptor (VLDLR) and apolipoprotein E receptor 2 (ApoER2) and low density lipoprotein receptor-related protein 1 (LRP1) independently of the presence of LDLR [72,73]. PCSK9 can also interact with the cluster of differentiation 36 (CD36) to reduce the uptake of free fatty acids and triglycerides [74]. CD81 (HCV receptor) levels are also regulated by PCSK9 [75]. It is also able to affect the levels of the intestinal cholesterol transporter NPC1L, likely indirectly via a transcriptional regulation mechanism [76].

## 4. PCSK9 in Cancers

There is growing evidence that PCSK9 expression is deregulated in various types of cancers and malignancies. Many published studies have revealed the altered expression of PCSK9 in primary cancers and in cancer cell lines as well as other malignancies.

### 4.1. Hepatocellular Carcinoma and Hepatoblastoma

In HCC tissue samples, immunohistochemical staining revealed that PCSK9 expression was downregulated along with an upregulation of the expression of LDLR as compared to adjacent cirrhotic tissues. This observation was confirmed using flash-frozen HCC samples where the mRNA level of PCSK9 was lower while that of LDLR was higher when compared to adjacent liver tissue as well as normal control tissue. However, the serum levels of PCSK9 in HCC patients were higher than in patients with chronic liver disease without HCC. Taken together, these data indicate that the microenvironment in HCC is modulated so as to have a constant energy supply in order to fuel tumor growth [77]. In contrast, Zhang et al. reported the high expression of PCSK9 in HCC, which not only correlates with poor prognosis of patients but also promotes the proliferation of cells in vitro as well as HCC progression in vivo [78].

A case report study involving a sixty-nine-year-old Japanese man with type III hyperlipoproteinemia (due to HBV infection)-associated HCC revealed an increase in the transcript levels of PCSK9 and its regulatory transcription factor sterol regulatory element binding protein 1 (SREBP2) in tumor tissues in comparison to non-tumor tissues. The serum level of PCSK9 was also increased. However, a massive decrease in the levels of LDLR and LRP1 was observed in the non-tumor tissues, suggesting that the increased SREBP2 expression triggers the increase and secretion of PCSK9, which in turn downregulates the lipoprotein receptors in the non-tumor tissue, hence resulting in paraneoplastic hyperlipoproteinemia [79].

In addition, there exists a link between PCSK9 and HCV infection, which is recognized as a cause for HCC development. Thus, it was shown that PCSK9 concentrations were significantly lower in HCV-Genotype-3 (G3) compared to HCV-Genotype-1 (G1). However, circulating PCSK9 levels did not correlate with LDL-C in HCV-G3 or G1. It was concluded that lower PCSK9 and LDL concentrations imply upregulated LDLR activity in HCV-G3 patients [80]. In another study, the plasma concentration of PCSK9 was significantly increased in chronic HCV-infected patients when compared to HCV-negative ones, but the genotypes were not defined. However, this HCV-induced increase in PCSK9 was independent from the presence of HCC [81].

More recently, we inhibited PCSK9 expression in three hepatoma (HCC and HB) cell lines, Huh6, Huh7 and HepG2. PCSK9 deficiency led to strong inhibition of cell proliferation in all cell lines (paper in revision). At the lipid metabolic level, PCSK9 inhibition led to excessive accumulation of intracellular lipids and a higher amount of lipid hydroperoxide. Molecular signaling analysis and morphological examination showed the disruption of p62/Keap1/Nrf2 antioxidative axis leading to iron-triggered cell death or ferroptosis (see below). The anti-tumoral effects of PCSK9 deficiency was validated using in vivo xenograft experiments in zebra fish.

### 4.2. Gastric Cancer

A stable isotope labeling by amino acids in cell culture (SILAC)-based quantitative proteomic analysis, which was carried out on the gastric cancer (GC) secretome, allowed the identification of 2205 proteins of which 263 proteins were highly expressed (>4-fold) when compared to non-neoplastic gastric epithelial cells. A 60% overexpression was observed for PCSK9, which was identified as one of the three candidate potential biomarkers for the early diagnosis and prognosis of GC [82]. In another study, the increased expression of PCSK9 was recorded in primary GC tissues vs. adjacent normal tissues, and high serum levels of PCSK9 was also reported in GC patients. This overexpression was associated with lymph node metastasis and poor prognosis [83].

### 4.3. Lung Cancer

A study investigating the mRNA expression levels of proprotein convertases in human lung cancer tumors in comparison to their matched adjacent tissues without pathology revealed a significant decrease in the expression of PCSK9 [84]. Another pilot study conducted in Italy involving patients with advanced non-small cell lung cancer (NSCLC) treated with Nivolumab (anti-programmed death 1 (PD1) monoclonal antibodies), revealed that those with high serum PCSK9 levels (>94 ng/mL) at the second cycle of nivolumab therapy had reduced overall survival (OS). As a result, high serum PCSK9 levels could serve as a prognostic marker in patients with advanced NSCLC [85,86].

Similar roles of PCSK9 have also been observed in other types of cancers, such as human neuroglioma, breast cancer, colorectal cancer and others [87]. This raises a major question about the potential therapeutic benefits of PCSK9 inhibition in these cancers.

## 5. PCSK9 Biological Functions

**Figure 3:**
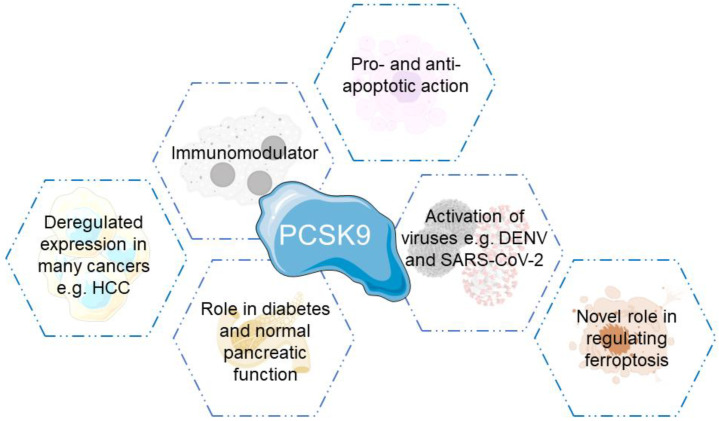
Biological functions and roles played by PCSK9.

PCSK9 has proven to be a key player in multiple physiological and pathological conditions, whether that includes cancer development, immunity or even normal organ function (Figure 3).

### 5.1. PCSK9 in Inflammation and Immunity

Inflammation is a critical process that contributes to tumor progression and aggressiveness. The role played by PCSK9 in inflammation has been extensively studied, especially in macrophages infiltrating TME, which are referred to as tumor-associated macrophages (TAMs). TAMs represent the most predominant immune cell population in the TME. The pro-inflammatory role of PCSK9 in atherosclerosis, i.e., an inflammatory disease involving many cytokines, was investigated by Tang et al. [88]. They revealed that the stimulation of THP-1 cells (human leukemic cell line 1) by oxLDL leads to the synthesis of high amounts of Interleukin-1 α (IL-1α), IL-6, tumor necrosis factor-α (TNF-α) and PCSK9. The process is regulated by an important transcription factor: the nuclear factor kappa-light chain-enhancer of activated B cells (NFƘB). OxLDL stimulation leads to the degradation of IƘBα, an inhibitor of NFƘB, resulting in the translocation of NFƘB to the nucleus and transcription activation. The inhibition of PCSK9 by small interfering RNA (siRNA) resulted in anti-inflammatory effects and blocked NFƘB [88]. Moreover, they further discovered that PCSK9 regulates inflammatory cytokine secretion by activating the Toll-like receptor 4 (TLR4)/NFƘB pathway. Silencing PCSK9 reduced the expression of inflammatory genes by blocking TLR4/NFƘB pathway in macrophages [89]. Moreover, Giunzoni et al. [90] showed that PCSK9-mediated inflammation in atherosclerosis was due to the high recruitment of inflammatory monocytes and their differentiation to macrophages. This pro-inflammatory action of PCSK9 was dependent on the presence of LDLR. The lipopolysaccharide (LPS)-induced differentiation of monocytes increased the expression of pro-inflammatory cytokines (IL-1β and TNF-α) and suppressed those of anti-inflammatory markers (arginase 1 (Arg1) and IL-10).

Furthermore, another group of scientists have confirmed the pro-inflammatory role played by PCSK9 on THP-1 derived macrophages, human primary macrophages and murine bone marrow-derived macrophages (BMM). They observed an induction in the expression of pro-inflammatory cytokines, such as IL-1β, IL-6, TNF-α and chemokines, such as CXCL2 (C-X-C motif chemokine ligand 2) and MCP1 (monocyte chemotactic protein-1) when these macrophages were incubated with human recombinant PCSK9. In addition, they revealed that this pro-inflammatory role of PCSK9 is mainly, but not strictly, dependent on the presence of LDLR, where they observed a high induction in TNF-α expression in BMM cells expressing LDLR (LDLR^+^/^+^) as compared to LDLR-deficient (LDLR^−^/^−^) cells. However, they still observed a significant effect in BMM-LDLR^−^/^−^ cells, which they suggested to be due to the participation of other receptors targeted by PCSK9, e.g., CD36, VLDLR, LRP-1 or ApoER2, in the pro-inflammatory response. Moreover, they demonstrated that the role played by PCSK9 was dependent on the activation of JAK and SREBP pathways as well as the nuclear translocation of p65 NFƘB transcription factor [91].

Very recently, LRP5 and PCSK9 were shown to participate in the lipid uptake and inflammation in human macrophages [92]. Badimon et al. revealed that LRP5 levels increase and translocate from the cytoplasm to the membrane whenever extracellular lipids are detected, and together with the lipids, they are internalized, resulting in the increased accumulation of cholesterol esters in human macrophages. PCSK9 expression was almost undetectable in monocytes, but it significantly increased in differentiating monocytes and its highest levels were found in fully differentiated macrophages. When human macrophages were loaded with lipids, this resulted in a significant decrease in PCSK9 protein levels, the increased secretion of PCSK9 into the medium and, most importantly, the perinuclear co-localization of both PCSK9 and LRP5. Immunoprecipitation experiments confirmed the intracellular interaction between PCSK9 and LRP5, which was found to be necessary for the PCSK9 release pathway. Because PCSK9 is known for its role in macrophage inflammation via the TLR4/NFƘB pathway, they further confirmed that inhibiting PCSK9 resulted in the downregulation of this pro-inflammatory pathway by decreasing the protein expression of TLR4 and the nuclear translocation of NFƘB. Moreover, PCSK9 silencing abrogated the increased expression and secretion of TNF-α and IL-1β, which is usually observed after lipid loading, hence supporting the role of PCSK9 in increasing human inflammatory signaling [92].

Interestingly, another study investigated the effect of pro-inflammatory cytokine TNF-α and Janus kinase/signal transducer and the activator of the transcription protein (JAK/STAT) pathway on the de novo lipogenesis and PCSK9 expression in the human HepG2 cell line. They showed that TNF-α induces the expression of PCSK9 by inducing the suppressor of cytokine signaling 3 (SOCS3), the endogenous inhibitor of STAT proteins. The inhibition of STAT3 by siRNA completely blocked the induction of SOCS3 and PCSK9 by TNF-α, hence validating the involvement of JAK/STAT pathway. On the other hand, overexpressing SOCS3 resulted in the inhibition of STAT3 phosphorylation, increased PCSK9 expression and induced de novo lipogenesis in HepG2 cells, which was reflected by the upregulation of key enzymes, such as fatty acid synthase (FAS), stearoyl-CoA desaturase (SCD-1) and apolipoprotein B (apoB) [93].

In melanoma, colon and breast murine cancer cells, the inhibition of PCSK9 synergistically boosted the tumor response to murine anti-PD1 immune checkpoint inhibitor, resulting in the suppression of tumor growth. Moreover, evolocumab (an inhibitory monoclonal antibody to PCSK9) treatment, alone or in combination with anti-PD1 treatment, also inhibited the tumor growth of cells resistant to immune checkpoint therapy. The depletion of PCSK9 in tumors increased the intratumoral infiltration of lymphocytes, such as CD8^+^ cytotoxic T-cells (CTLs), CD4^+^ T helper (T_h_) cells, γδT cells and natural killer (NK) cells, thus rendering the tumor responsive to immune checkpoint therapy. The molecular mechanism by which CTL killed PCSK9-deficient tumor cells involved an increase in the tumor cell surface MHC I (Major Histocompatibility Complex I) expression in PCSK9-deficient tumors as compared to control, indicating that PCSK9 downregulates MHC I surface levels by lysosomal degradation in a manner very similar to the downregulation of LDLR protein levels [94].

Another recent study carried out by Yuan et al. [95] investigated the critical role of metabolic regulation in T cell antitumor activity. Their study revealed the importance of LDLR/PCSK9 axis in regulating CD8^+^ T cell antitumor activity. Indeed, they discovered a non-canonical function of LDLR in CD8^+^ T cells, in which this receptor interacts with the T-cell receptor (TCR) complex to regulate its recycling and signaling, hence facilitating cytotoxic T-lymphocytes’ (CTLs) effector function. In addition, they also revealed the importance of tumor microenvironment in affecting the function of CTLs, where elevated levels of PCSK9 by tumor cells allows its binding to LDLR and prevents the LDLR/TCR complex from being recycled back to the cell surface, resulting in decreased levels of LDLR on CD8^+^ T cells and decreased TCR signaling, leading eventually to the inhibition of the effector function of CTLs. The genetic or pharmacological targeting of PCSK9 in tumor cells enhances CD8^+^ T cells’ antitumor activity as well as tumor progression. Their study highlighted the LDLR/PCSK9 axis as a potential target for future cancer immunotherapies.

### 5.2. PCSK9 in Diabetes

PCSK9 is necessary for the normal function of the pancreas because it is expressed by insulin-producing pancreatic islets β-cells [34] along with LDLR. Using both whole body knockout (KO) or β-cells specific KO models, it was recently demonstrated that PCSK9 deletion in mice does not have any toxic effect on β-cell function and does not affect insulin levels or glucose homeostasis [96]. Another study has revealed the role played by insulin in regulating PCSK9 both in vitro and in vivo [97]. Insulin increased the expression of PCSK9 and in turn the degradation of LDLR. PCSK9 levels were decreased markedly (up to 80%) in mice knockout for insulin receptor or treated with streptozotocin or antisense oligonucleotides to inactivate their insulin receptors. These observations can be explained by an additional mechanism involving glucagon to influence PCSK9 levels. Glucagon decreased the mRNA and protein levels of PCSK9 by 50% in primary rat hepatocytes, along with decreasing the transcription of SREBP-1c and SREBP-2 by 20-50%. In addition, LDLR levels were also diminished by 20% at the mRNA levels, whilst their protein level was increased by two-fold.

Another pathway that regulates PCSK9 levels was identified by Ai et al. [98]. During early stages of diabetes and type 2 diabetes, high LDL turnover is present and is dependent on hyperinsulinemia. In this study, the authors observed that obese mice suffering from hyperinsulinemia had 60% lower mRNA levels of PCSK9 and increased LDLR. This was due to the fact that insulin activates the mammalian target of the rapamycin complex 1/protein kinase Cδ (mTORC1/PKCδ), which then inactivates hepatocyte nuclear factor 1 α (HNF1α) and blocks PCSK9 transcription. Hence, the mTORC1 pathway herein links diabetes to lipid metabolism.

### 5.3. PCSK9 in Apoptosis

Apoptosis is a genetically regulated form of cell death that involves many molecular mechanisms. The ability of a cell to undergo mitochondrial apoptosis is governed by the ratio of pro-apoptotic to anti-apoptotic proteins: BCL2 associated X protein/B-cell lymphoma 2 (Bax/Bcl-2). Bax proteins are known for their pro-apoptotic effect by perforating the outer mitochondrial membrane and increasing its permeability, while Bcl-2 is a family of anti-apoptotic proteins that inhibit apoptosis [99]. A strong correlation between PCSK9 overexpression and increased oxLDL-induced apoptosis in vascular endothelial cells was observed [37]. This was further confirmed by inhibiting PCSK9 using siRNA, which led to the reduction of the Bcl-2/Bax ratio and the inhibition of caspase 9 and 3, hence decreasing apoptosis [100].

In neurons, PCSK9 was also found to regulate the neuronal apoptosis through the c-Jun N-terminal kinase (JNK) pathway by adjusting ApoER2 levels and signaling [101]. The inhibition of PCSK9 showed an anti-apoptotic effect on cerebellar granule cells (CGN) due to the reduction in nuclear phosphorylated, active, c-Jun and activated caspase 3. This was also accompanied with higher ApoER2 protein levels. The knockdown of ApoER2 reversed the action of PCSK9 inhibition, suggesting that the PCSK9-mediated degradation of ApoER2 serves as a factor in the JNK pathway and phosphatidylinositol 3 kinase (PI3K) and extracellular signal-regulated kinase 1/2 (ERK1/2) activity. In another study performed on brain glioma cells U251, the opposite action of PCSK9 was found. The siRNA inhibition of PCSK9 in U251 cells promoted apoptosis through caspase 3 activation and the decrease of anti-apoptotic proteins: X-linked inhibitor of apoptosis protein (XIAP) and phospho-serine/threonine kinase (pAKT) and an increase in the ratio of Bax/Bcl-2 and cytochrome c distribution to the cytosol, whilst the overexpression of PCSK9 inhibited the apoptosis and decreased Bax/Bcl-2 ratio [102].

In human lung adenocarcinoma cells (A549), PCSK9 was shown to regulate apoptosis through ER stress (ERS) and mitochondrial signaling pathways [103]. The use of PCSK9 siRNA resulted in an anti-tumor activity by the induction of apoptosis. This was possibly due to the activation of caspase-3 and the downregulation of the anti-apoptotic proteins survivin and XIAP. In addition, an increase in Bax/Bcl-2 ratio was also recorded, leading to the release of cytochrome c after PCSK9 siRNA transfection. The ERS was also increased because of an increase in the levels of glucose related-proteins (GRP78 and GRP94), phosphorylated protein kinase R-like ER kinase (p-PERK) and phosphorylated eukaryotic initiation factor 2 α(p-eIF2α), leading to cell death in A549 cells.

In prostate cancer (PCa), the inhibition of PCSK9 using siRNAs protected the PCa cells from ionizing radiation (IR)-induced cell damage. The IR-exposed PCa cells showed a decrease in cell viability along with an increase in apoptosis, which can be reflected by the increase in cytochrome c, caspase-3 and Bax/Bcl-2 ratio. All of these were reversed when the PCa cells were treated with siRNA against PCSK9 before IR, indicating that PCSK9 is capable of affecting the mitochondrial membrane stability and that PCSK9 siRNA can induce radio-resistance through mitochondrial signaling pathways [104]. In gastric cancer (GC), the overexpression of PCSK9 plays an important role in the development of this disease where it contributes to increased tumor aggressiveness, which is reflected by increased migration and invasion and decreased apoptosis. Moreover, the signaling pathway by which PCSK9 promotes GC metastasis and suppresses apoptosis involves the activation of mitogen-activated protein kinase (MAPK) pathway through the upregulation of heat shock protein 70 (HSP70) expression [83]. In addition, in cases of the liver metastasis of melanoma cells (B16F1), mice lacking the *PCSK9* gene showed lower levels of metastasis when on a chow diet. This effect was absent when feeding mice a high-fat diet, revealing that high cholesterol levels promote metastatic progression. The lack of PCSK9 is associated with higher apoptosis in liver stromal cells due to the increased expression of TNF-α and its receptor (TNFR1) and decrease in Bcl-2 [105].

In HCC, the high expression of PCSK9 not only correlates with the poor prognosis of patients, but it also promotes the proliferation of cells in vitro as well as HCC progression in vivo. This is due to the inhibition of apoptosis of HCC cells exerted by PCSK9 via the fatty acid synthase (FASN)/Bax/Bcl-2/Caspase9/Caspase3 pathway [78]. On the other hand, in another study using different HCC cell lines (HepG2 and LM3), PCSK9 expression was enhanced using acRoots (*Actinidia chinensis* Planch root extract), resulting in decreased LDLR expression, the inhibition of LDL uptake by LM3 cells, the decrease in the intracellular cholesterol levels and thus diminished proliferation. These effects were confirmed using two approaches: PCSK9 overexpression and PCSK9 knockdown [106]. The contradiction we observe in the role of PCSK9 with respect to apoptosis necessitates further examination to examine whether it is indeed promoting or suppressing proliferation and apoptosis in HCC and whether this is dependent on the HCC genotype.

### 5.4. PCSK9 in Viral Infections

It is not strange to find a connection between proprotein convertases and viral infections. Indeed, an important role for PCs, such as furin, SKI-1/SIP and PCSK9, has been reported as activating a number of enveloped viruses, such as dengue virus (DENV) and SARS-CoV-2 [46]. Hepatocytes infected with DENV led to the increased mRNA expression of PCSK9, reduced LDLR protein levels and LDL-C uptake, resulting in increased cholesterol de novo synthesis [107]. Moreover, patients with dengue have elevated plasma levels of PCSK9, which is strongly and positively correlated with high levels of viremia. Targeting PCSK9 using monoclonal antibody (alirocumab) reversed this observation, where higher LDLR levels and lower viremia were recorded. This highlights the possible antiviral therapeutic strategy of inhibiting PCSK9, which still needs to be further studied, especially at the clinical level, before being introduced as a treatment option for DENV-infected patients [38]. In the context of SARS-CoV-2, lipid-lowering drugs, such as statins and PCSK9 inhibitors, have been tested. Seidah et al. [108] summarized the novel outcomes of such treatments. Briefly, the combination of statins with PCSK9 inhibitors has been suggested as an efficient treatment for improving the prognosis of COVID-19 patients in two ways: (a) effectively decreasing LDL-C levels and (b) preventing the reduction of antiviral genes expression, specifically that of interferons [109].

### 5.5. PCSK9 in Ferroptosis

Death is the ultimate fate of life, for organisms and cells. Cell death that is regulated by dedicated molecular machinery is known as regulated cell death (RCD). This means that it can be adjusted (delayed or accelerated) by certain pharmacological or genetic interventions. RCD is used to describe the death of cells caused by the fluctuations of the intracellular or extracellular microenvironment; when such fluctuations are too strong or prolonged, the adaptive responses are unable to restore cellular homeostasis [110,111]. Ferroptosis is one form of RCD that has been observed many times over the years, but it acquired such terminology in 2012 [112]. Briefly, ferroptosis is an iron-dependent and lipid reactive oxygen species (ROS)-reliant non-apoptotic mode of cell death with distinct morphological changes, including small mitochondria with increased mitochondrial membrane densities, decreased or disappeared mitochondria crista, the rupture of outer mitochondrial membrane and a normal nucleus [112,113]. The accumulation of lipid-based ROS becomes lethal when the antioxidant defense systems such as glutathione (GSH)-dependent lipid peroxide repair systems are compromised. This is why lethal lipid peroxidation, especially that of polyunsaturated fatty acyl phospholipids (PUFA-PLs), lies at the center of ferroptosis [114]. Figure 3 summarize the most important molecular pathways that regulate the induction or inhibition of ferroptosis. It is important to note that cell death by ferroptosis can also be regulated by multiple compounds or drugs which can either induce or inhibit ferroptosis [113,115].

With respect to proprotein convertases, especially PCSK9, their effect on ferroptosis remains unknown. In fact, it was only until 2021 when a group of scientists discovered a link between furin (the third member of PC family [116]) and ferroptosis in ulcerative colitis (UC) [117]. In this study, DSS (dextran sodium sulfate)-treated NCM460 cells (human normal colonic epithelial cell line) were used as an in vitro model of colitis. They revealed that the overexpression of furin in these cells attenuated DSS-induced ferroptosis-like injury and increased the expression and nuclear localization of Nfr2, which in turn upregulated the expression of a downstream gene: GPX4. On the contrary, the siRNA silencing of furin resulted in opposite effects. Similar findings were obtained in vivo using DSS-induced colitis mice model, where treating these mice with exogenous furin relieved DSS-induced colitis, a mechanism involving the activation of Nrf2 and the upregulation of GPX4. Therefore, this study concludes that furin exerts a protective effect on epithelial cells suffering from experimental colitis by inhibiting ferroptosis-like cell injury via a mechanism involving the activation of the Nrf2-GPX4 signaling pathway. Nonetheless, the authors still confirm the limitations that this study has, indicating that further investigations are needed [117].

Interestingly, searching the web for any connection between PCSK9 and ferroptosis was unsuccessful. Luckily enough, we are probably the first to find a connection between inactivating PCSK9 in liver cancers and the induction of ferroptosis [118]. In summary, the genetic inhibition of PCSK9 in liver cancer cells resulted in a decrease in cell viability and migration, an increase in the levels of intracellular lipids and led to high levels of lipid hydroperoxide. The molecular mechanism by which cell death was induced in liver cancer cells involved the disruption of p62/Keap1/Nrf2 antioxidative axis leading to ferroptosis. Indeed, ferroptosis induction was further confirmed by the morphological changes that we observed in the mitochondria (shrinkage, vanished crista, thickened membrane) using electron and confocal microscopies. Taken together, we have unveiled a novel function of PCSK9 as a new modulator of ferroptosis.

These investigations highlight the important role played by PCs, in this case furin and PCSK9, as protective proteins against ferroptosis induction. However, further investigations are needed, especially at the molecular level, to confirm this intriguing connection. Nonetheless, the present results suggest that inhibition/silencing PCSK9 could potentially be considered as a promising anti-cancer approach to treat liver cancers.

## 6. PCSK9 Targeting as a Potential Anti-Cancer Approach

The important role of PCSK9 in lipid homeostasis brings to the fore an attractive target for many diseases, such as FH, cardiovascular disease and especially cancer. Different studies involving cancer animal models and cancer cell lines showed the high potentiality of PCSK9 as a possible therapeutic target for many diseases. So far, we observed a high expression of PCSK9 in the majority of cancers, suggesting that targeting it could serve as an effective anticancer strategy. Several approaches can be used to target and inhibit PCSK9, including (a) blocking the binding of PCSK9 to LDLR using monoclonal antibodies (mAbs), anti-PCSK9 vaccines, adnectins, mimetic peptides or novel molecules; (b) inhibiting the expression of PCSK9 using CRISPR/Cas9 genome-editing tool, antisense oligonucleotides (ASOs), siRNA or recently discovered pharmaceutical drugs (R-IMPP or PF-06446846, etc.); or (c) interfering with the secretion of PCSK9 from the ER by sortilin or Sec24a (Table 1).

### 6.1. Blocking PCSK9 Binding to LDLR

This is the first approach used to inhibit PCSK9 by adopting multiple strategies:

#### 6.1.1. PCSK9 Monoclonal Antibodies (mAbs)

This strategy uses humanized mAbs that can recognize and bind the catalytic domain of PCSK9, thereby blocking its interaction with the EGF-A domain of LDLR. Due to this, PCSK9 fails to bind LDLR and the latter can escape degradation [150]. This approach has been extensively studied in many clinical trials (Phase I to III), alone or in combination with other lipid lowering drugs like statins [119]. Two fully humanized anti-PCSK9 mAbs are currently approved for the treatment of hypercholesterolemia: (i) evolocumab, (AMG145, trade name Rephata) developed by Amgen (Amgen, Thousand Oaks, CA, USA) and approved by the European Commission in July 2015 and the FDA one month later, and (ii) alirocumab (SAR236553/REGN727, trade name Praluent), developed by Regeneron and Sanofi Pharmaceuticals (Regeneron Pharmaceuticals, Inc., Tarrytown, NY, USA; Sanofi, Paris, France) and approved by Endocrinology and Metabolic Drugs Advisory Committee (EMDAC) of the US FDA in July 2015. These drugs have enabled a marked reduction in LDL-C levels in dyslipidemic patients. Several trials and meta-analyses showed a ca. 50–60% reduction in LDL-C levels using these mAbs in patients with a diet-based therapy, whether alone or in combination with different doses of statins, without any serious side effects [119,120,122].

Evolocumab was clinically evaluated in combination with statin treatment in 27,564 high-risk patients with atherosclerotic cardiovascular disease (FOURIER study; ClinicalTrials.gov number: NCY01764633). In this clinical trial, the levels of LDL-C were decreased by 59% using the combination treatment along with 15% decrease in the cardiovascular events (cardiovascular death, strokes, myocardial infarction, hospitalization for unstable angina or coronary revascularization) after a 2.2-year follow-up [121]. On the other hand, alirocumab was also evaluated in clinical trials in 18,924 patients who had acute coronary syndrome and high LDL-C levels despite of receiving high doses of statins (ODYSSEY OUTCOMES; ClinicalTrials.gov number: NCT01663402). LDL-C levels were decreased by 61% compared to placebo after one year, although around 90% of patients were taking high doses of statin. Moreover, adverse cardiovascular events were also reduced by 15%, including CHD death, non-fatal myocardial infarction, non-fatal ischemic stroke and hospitalization for unstable angina [123]. Taken together, both of these studies show the effectiveness of PCSK9 mAbs in high-risk patients with LDL-C > 70 mg/dL despite the use of high doses of statin.

Aside from these promising results, there are still some limitations for the use of mAbs. First, they need to be taken through a subcutaneous injection 1–2 times per month. Their price is quite high, given their estimated cost of USD 5000–7000 per person per year in Europe. However, it should be noted that the dose frequency of these injections is low compared to other types of injections, such as insulin. Nonetheless, improving the cost effectiveness of these mAbs is necessary to provide better treatment options for a wide range of populations [58].

#### 6.1.2. Anti-PCSK9 Vaccine

Due to the many limitations associated with mAbs, active vaccination is useful for providing better efficiency and lower cost therapeutic approaches. The Nanoliposomal anti-PCSK9 L- vaccine (L-IFPTA^+^: Liposomal Immunogenic Fused PCSK9-Tetanus plus Alum adjuvant) has been recently developed and has resulted in an efficient and safe induction of long-lasting PCSK9-specific antibodies in vaccinated BALB/c mice. The vaccine-stimulated Abs disrupted the function of PCSK9 by blocking its interaction with LDLR [124,151]. It also protected against hypercholesterolemia and atherosclerosis in C57BL/6 mice [125,126]. Moreover, the L-IFPTA^+^ vaccine was shown to be immunogenic and safe in healthy non-human primates in a pre-clinical study, suggesting that it could be eligible for a phase I clinical trial in humans [127]. With respect to cancer, mice bearing breast cancer that were vaccinated with the L-IFPTA^+^ vaccine showed a moderate, but not significant, decrease in tumor growth (21.2%) and a prolonged lifespan by 4.2%. Additionally, the survival in the combination and chemotherapy with Doxil groups was significantly higher than the vaccine and control groups. Unfortunately, this peptide-based (aa 153–165 of PCSK9) vaccine only reduced the levels of free mouse PCSK9 in plasma by 50%, and hence was far from being as efficient as the mAb treatment. Nevertheless, this reveals that the inhibition of PCSK9 by vaccination had no side effects on the breast tumor endpoint and that it could improve breast cancer behavior [152]. On the other hand, vaccination with L-IFPTA^+^ vaccine in mice bearing melanoma did not show any effect on tumor growth nor the survival of tumor-bearing mice [153]. Here again, the same peptide-based vaccine only bound 50% of circulating mouse PCSK9. Taken altogether, vaccination seems to be an interesting strategy to inhibit PCSK9, but it clearly has to be optimized with better epitopes and vaccination strategies to be reliably effective. Thus, more studies and clinical research should be undertaken to investigate and enhance the efficacy of the vaccine in humans and to evaluate whether a highly potent vaccine is indeed effective in inhibiting tumor growth in other cancers.

#### 6.1.3. Adnectin

Adnectins are small (~12 kDa) synthetic proteins based on the 10th type III domain of human fibronectin, whose variable loops can be designed to bind therapeutic targets with high affinity and specificity. They possess a β-sheet fold structure with diversified loops similar to Ab variable domains; however, they differ from Abs in primary sequence and have a single domain structure lacking disulfide bonds [154]. Adnectin, BMS-962476 by Bristol-Myers Squibb/Adnexus, is a PCSK9-binding polypeptide engineered with polyethylene glycol to enhance pharmacokinetics and bind with a subnanomolar affinity to human PCSK9 [128]. Adnectin impedes the interaction between the PCSK9 and EGF-A domain of LDLR, thus preventing its degradation, similarly to mAbs. The production of adnectin is less expensive and easier than mAbs, since it can be produced using bacterial expression systems, thereby serving as a potential alternative to mAbs. In a preclinical trial carried out on cynomolgus monkeys, BMS-962476 (5 mg/kg) decreased the free PCSK9 levels by >99% and LDL-C by ~55% within 48 h, followed by a six-fold increase in total PCSK9 [128]. In humans, the single ascending subcutaneous (SC) or intravenous (IV) doses of BMS-962476 resulted in a >90% decrease of free PCSK9 along with a ca. 48% decrease in LDL-C levels at maximal dose [129], without any serious adverse event.

Taken together, these observations suggest that BMS-962476 is a promising substituent drug for mAbs that can target circulating PCSK9 and significantly decreases LDL-C levels. Nevertheless, its effects should be followed on a larger scale and over long periods of time to make sure of its efficacy and safety. However, with the clinical success of the mAb and siRNA treatments, it seems that the adnectin approach has been abandoned.

#### 6.1.4. Mimetic Peptides

They are small amino acid (aa) sequences designed to biologically look like the peptidic structure of a target protein. Peptides mimicking the EGF-A or EGF-AB binding domains of LDLR were designed as competitive inhibitors of PCSK9 that bind to the catalytic domain of PCSK9, thus preventing its interaction with LDLR. They are able to serve as therapeutic alternatives to small molecules and large Abs [155]. For instance, a truncated 26-aa EGF-A analog was recently synthesized which served as an inhibitor for PCSK9-LDLR binding. This peptide exhibited a high binding affinity for PCSK9, leading to the increased recycling of LDLR and thus the lowering of LDL-C levels [156]. Moreover, another small peptide that mimics the secondary structural elements of the EGF-A domain called Pep2-8 was discovered to attenuate the interaction between PCSK9 and LDLR, leading to the full restoration of LDLR surface levels and the uptake of LDL-C by HepG2 cells treated with PCSK9 [130].

Annexin A2 (AnxA2) is a natural extrahepatic inhibitor that binds to the C-terminal domain of PCSK9, preventing its interaction with LDLR and hence PCSK9-mediated degradation. It is abundant in lung, pancreas, colon and adrenal glands, while lower levels are found in the liver, kidney and spleen. AnxA2 knockout mice show a ~2-fold increase in the circulating PCSK9 and ~1.4-fold increase in LDL-C levels, along with a ~50% decrease in LDLR protein levels in extrahepatic tissues. However, the overexpression of AnxA2 in liver leads to an increase of LDLR proteins [131]. Thus, the inhibitory role of AnxA2 in PCSK9 modulates the degradation of LDLR, and for that reason, creating small molecules that mimic AnxA2 could be a good approach for PCSK9 inhibition.

#### 6.1.5. Pseurotin A

Pseurotin A (PS) is a unique spiro-heterocyclic γ-lactam alkaloid isolated from the fungus *Aspergillus fumigatus*. It has an anti-inflammatory role and anti-seizure activity and has been shown to be a potential treatment and preventive for osteoporosis. Interestingly, in a very recent study, PS was shown to inhibit the expression and secretion of PCSK9 in hepatic cancer cells, HepG2 and Huh7, respectively. The mechanism by which PS exerts its function is through binding to the catalytic domain of PCSK9 (which usually accommodates the EGF-A domain of LDLR), thus inhibiting its interaction with LDLR. Moreover, PS also showed an anti-proliferative effect in breast cancer cell lines (BT-474 and T47D) and resulted in reducing PCSK9 levels while increasing LDLR levels in a dose-dependent manner. Similarly, in vivo experiments confirmed such observations, where PS treatment in orthotopic nude mice bearing BT-474 tumor cells xenograft model fed a high fat diet (HFD) resulted in a more than 59% reduction in tumor growth, reduced the expression level of PCSK9 and decreased the level of circulating cholesterol as compared to vehicle control treatments. Finally, PS inhibited the locoregional tumor recurrence by decreasing PCSK9 levels [132]. This interesting study has brought PS, a fungal metabolite, to the spotlight as a novel small molecule that targets PCSK9 and prevents tumor growth in vitro and in vivo. However, more research is needed to better understand the mode of action of PS in many cancer models.

#### 6.1.6. Silibinin A

Due to the high cost of FDA-approved monoclonal antibodies targeting PCSK9, the development of a low-cost alternative is a necessity. Traditional Chinese medicine (TCM) is an answer to low-cost effective treatments that has been in clinical practice for a long time. Silibinin (SIL) is a polyphenolic compound that belongs to the flavonoids and represents the main bioactive ingredient of silymarin (silybin), a standard extract of milk thistle seeds [157]. It has been clinically used to treat liver diseases, such as acute and chronic hepatitis, early cirrhosis and poisonous liver injury [133]. SIL was discovered to effectively decrease the promoter activity of *PCSK9* as well as the mRNA and protein levels in a dose/time-dependent manner in HepG2 cells. Moreover, the statin-induced increase of PCSK9 expression was attenuated when SIL was co-incubated with atorvastatin in HepG2 cells. This effect was mediated by suppressing the p38 MAPK pathway, thus enhancing the lipid-lowering activity of statin [134]. However, further studies are needed to confirm this additive effect of SIL along with statin, especially in vivo.

#### 6.1.7. Orally Active Tricyclic Peptide MK-0616

The tricyclic peptide MK-0616 that exhibits picomolar potency against PCSK9 was formulated with permeation enhancers to enable once-daily oral delivery [135]. MK-0616 reduced LDL-C without causing serious side effects or death when taken as once daily oral doses of up to 300 milligrams alone in healthy men, or in combination with statins among men and women with high cholesterol. MK-0616 lowered LDL-C by approximately 65% from baseline levels after 14 days of treatment. Patients treated with the placebo had a less than 5% reduction in LDL-C compared to baseline. Thus, MK-0616 is a potent oral PCSK9 inhibitor that effectively lowers LDL-C as shown in Phase I and ongoing Phase II clinical trials [136].

### 6.2. Inhibition of PCSK9 Expression

Different approaches can be used in this strategy, including genomic and pharmaceutical methods:

#### 6.2.1. CRISPR/Cas9 Genome-Editing Tool

Clustered Regularly Interspaced Short Palindromic Repeat (CRSIPR)/CRISPR-Associated Protein 9 (Cas9) is a very useful genome-editing tool that is used to destroy or edit a target gene. It is an accurate, fast and cheap editing system. It consists of Cas9 endonuclease and a single guide RNA (sgRNA) which has, at its 5′ end, a 20-nucleotide sequence capable of recognizing and binding with a complementary sequence on the target DNA. Cas9 can induce DNA cleavage, generating a double strand break and triggering DNA repair machinery by non-homologous end-joining (NHEJ) or homology-directed repair (HDR). NHEJ is error-prone, resulting in disruptive insertions and/or deletions at the target site, while HDR can use an exogenous DNA repair template to induce a knock-in of the desired alteration in the genome [158]. Several studies have been successfully able to abrogate the *PCSK9* gene using this tool. As a result, a significant reduction in plasma PCSK9 levels was observed with an increase in hepatic LDLR levels. The plasma LDL-C levels were decreased by 35–40% [159,160,161].

Most known pathogenic point mutations in humans are C•G to T•A substitutions, which can be directly repaired by adenine base editors (ABEs). Thus, more recently, a single infusion of lipid nanoparticles allowed the delivery of a more advanced base-editing CRISPR-ABE system that successfully modified the *PCSK9* gene of cynomolgus monkeys and achieved ~90% and ~60% reductions of plasma PCSK9 and LDL-C, respectively, for more than 8 months [137]. A similar approach was also used in macaques [138].

This means that a tool, such as CRISPR/Cas9, can be used to permanently alter the human genome to prevent for instance CHD in hypercholesterolemic patients. However, many ethical, legal and safety issues need first to be resolved before integrating it as a therapeutic approach in humans [58], since it is associated with several limitations, such as off-target mutations.

#### 6.2.2. Antisense Oligonucleotides (ASOs)

These are short, single-stranded oligonucleotides 12–25 base pair long that bind directly to a target mRNA by Watson–Crick base pairing in order to block its translation in cytosol and nucleus. The degradation of mRNA by these ASOs depends on the activity of the RNase H enzyme, which recognizes RNA-DNA heteroduplexes [162]. 2′-O-methoxylethyl RNA (ISIS 394814/BMS 844,421 by Bristol Myers Squibb/ISIS Pharmaceutical) is a second-generation ASO that targets PCSK9 mRNA evaluated in preclinical studies. High doses of this ASO lead to a decrease in PCSK9 mRNA levels by 92% and an increase in LDLR protein levels by two-fold, resulting in a 38% reduction of LDLD-C levels in vivo [139]. However, the development of this family of ASOs was halted due to their low binding affinity. Later, a new generation of shorter ASOs (SPC4061/SPC5001 by Santaris Pharma A/S) was developed, with better stability, stronger affinity and specificity to PCSK9 mRNA. These ASOs have proven to be effective 24 h post-injection with a 60% reduction in PCSK9 levels, persisting up to 16 days after treatment [140]. In another study, the subcutaneous injection of SPC5001 decreased PCSK9 and LDL-C levels by 50% and 25%, respectively and dose-dependently, while also decreasing ApoB and increasing ApoA1 levels [141]. As promising as they seem, they caused acute kidney injury in one patient, which led to termination of clinical development [142].

However, it is important not to neglect the potential therapeutic benefit of ASOs, which needs much development in order to reduce adverse effects by making them accurately match the target mRNA and be delivered specifically to the desired cells to minimize off-target toxicity.

#### 6.2.3. siRNA

Small interfering RNA (siRNA) is a synthetic RNA duplex that targets mRNA for degradation or translation silencing, thereby inducing a gene knockdown. Frank-Kamenetsky et al. [163] have developed siRNAs in lipidoid nanoparticles that can target PCSK9 in several species (murine, rat, non-human primate (NHP) and human) and validate their efficacy in vivo. They found a 50–70% decrease in PCSK9 mRNA levels in mice and rats. This was accompanied by a 60% decrease in LDL-C levels. Furthermore, transgenic mice expressing human PCSK9 also showed a >70% decrease in PCSK9 transcript and plasma levels upon siRNA administration. In NHP, similar observations were seen and the effects lasted for 3 weeks after a single intravenous injection.

Inclisiran (Leqivo^®^; Novartis) is the first-in-class chemically synthesized siRNA conjugated to N-acetylgalactosamine carbohydrate (GalNAc) to target ASGR1 in liver hepatocytes [164] designed to decrease cholesterol levels [165]. In December 2020, Europe gave inclisiran its first approval for the use in adults suffering from heterozygous FH or non-familial and mixed dyslipidemia [143,144]. Inclisiran targets PCSK9 mRNA and, because it is conjugated to GalNAc, this will allow the precise and targeted uptake of the drug by hepatocytes. It is used in a combination treatment with statin or other lipid-lowering drugs in the case of statin-intolerant patients. The mode of administration of inclisiran is via a subcutaneous injection, which delivers an equivalent dose of 284 mg in 1.5 mL solution. It is recommended to receive a single shot on day 1, day 90 and then every 6 months [144]. The efficacy in a long-term reduction in the plasma PCSK9 levels, and hence reduction in LDL-C, is confirmed by all three phases III ORION studies (ORION-9, ORION-10 and ORION-11) in patients with heterozygous FH [145].

It is worth mentioning that a new drug application for inclisiran in patients with FH and atherosclerotic cardiovascular disease (ASCVD) was presented in December 2019 to the US FDA; however, a delay occurred due to the 2019 coronavirus disease (COVID-19)-related travel restrictions [146,147].

#### 6.2.4. Pharmaceutical Drugs

Some studies have checked the capacity of small pharmaceutical molecules in inhibiting PCSK9 expression through a mechanism involving the stalling of human 80S ribosomal subunit during translation. Two molecules have been discovered, R-IMPP and PF-06446846, which suggest the therapeutic potential behind the use of selective inhibitors of mRNA translation.

R-IMPP ((R)-*N*-(isoquinolin-1-yl)-3-(4-methoxyphenyl)-*N*-(piperidin-3-yl) propanamide) is a small-molecule compound identified using phenotypic screening to inhibit PCSK9 secretion [148]. It is an anti-secretagogue of PCSK9 by Huh7 cells that endogenously express PCSK9, leading to higher LDLR surface levels and the increased uptake of LDL. Since it did not affect the secretion of transferrin by Huh7 cells, this suggests that it does not exert its action via secretion inhibition. However, the specificity by which R-IMPP blocks PCSK9 protein synthesis is by binding to the human 80S ribosomal subunit and a sequence at the N-terminus (signal peptide and a portion of prodomain) of PCSK9 mRNA, resulting in the translation inhibition of PCSK9 mRNA. Yet, R-IMPP can also have off-target effects on other proteins, which may or may not play a role in modulating PCSK9 levels. The discovery of this molecule provides a new approach to target the synthesis of PCSK9 in the context of translating ribosomes.

PF-06446846 (by Pfizer) is the first example of an orally-active small molecule that is capable of reducing the synthesis of PCSK9, hence “inhibiting its function” [166]. It was able to reduce plasma PCSK9 and total cholesterol levels in vivo without any sign of toxicity in the liver of rats. However, this does not exclude the fact that the translation of a very small number of transcripts was also affected by PF-06446846, leading to certain adverse effects outside the liver at elevated doses. The mechanism of action of PF-06446846 is based on ribosome stalling on the PCSK9 transcript a few codons beyond to the end of signal peptide, leading to a direct and selective inhibition of PCSK9 translation during the elongation phase [149].

However, more experiments need to be performed to further optimize both of these molecules in order to improve their selectivity for PCSK9 only with no off-target effect on other essential proteins.

### 6.3. Interfering with PCSK9 Secretion

PCSK9 is synthesized and transported from ER to the Golgi complex and finally secreted into circulation. Its secretion can be inhibited by some LOF mutations in the prodomain or even targeting molecules, such as sortilin or Sec24a.

#### 6.3.1. Sortilin

*SORT1* gene encodes sortilin that is a high affinity sorting receptor of PCSK9. It co-localizes with PCSK9 in the trans-Golgi network and facilitates its secretion to the plasma membrane. This type of interaction is pH-dependent and at pH 6.5 (in trans-Golgi), there is a strong interaction between PCSK9 and sortilin, while at pH 5.5 (endosomes), PCSK9 is released. Any deficiency in sortilin expression leads to the increased intracellular levels of PCSK9 and decreased that of circulating PCSK9. Sortilin overexpression increases circulating PCSK9, reduces LDLR and increases LDL-C levels. All of these indicate a positive correlation between sortilin and PCSK9 [167]. Thus, targeting sortilin could be a useful tool to deregulate the levels of PCSK9. However, another study showed that silencing sortilin had no effect on endogenous and exogenous PCSK9 function on the LDLR both ex vivo and in mice [168].

#### 6.3.2. Sec24a

Sec24a is another protein required for the transport of PCSK9 from ER to the Golgi apparatus. Observations similar to those for sortilin have been made regarding Sec24a deficiency or overexpression [58], which is why it could also serve as a potential therapeutic target.

## 7. Conclusions

Beyond its critical role in maintaining lipid homeostasis, PCSK9 is implicated in various signaling pathways, including antiviral activity, apoptosis and, more recently, anti-tumor immune responses, as well as anti-oxidative housekeeping activities. Considering all these anti-tumoral effects of anti-PCSK9 approaches and taking into account the existence of a wide variety of therapeutic strategies of PCSK9 blockade (monoclonal antibodies, small molecule and peptide inhibitors, antisense oligonucleotides, siRNA, etc.), the expectations are high in terms of seeing this multifaceted protein as a very valuable and attractive target for the future potential treatment of liver cancers.

## Figures and Tables

**Table 1:** List of drugs or molecules tested as inhibitors of PCSK9, some of which are already FDA-approved.

Targeting Strategy	Drug/Approach	Type of Drug/Approach	Development Stage	Reference
Blocking PCSK9 binding to LDLR	Evolocumab (Rephata)	mAb	FDA-approved for treating hypercholesterolemia +/− Statins	[119,120,121]
	Alirocumab (Praluent)	mAb	FDA-approved for treating hypercholesterolemia +/− Statins	[119,122,123]
	L-IFPTA+	Nanoliposomal vaccine	Tested in vivo on mice Pre-clinically tested in non-human primates	[124,125,126] [127]
	BMS-962476	Adnectin	Pre-clinical trial carried out on cynomolgus monkeys Clinically tested in humans	[128] [129]
	Pep2-8	mimetic peptides	Tested in vitro	[130]
	Annexin A2	mimetic peptides, natural inhibitor	Tested in vivo	[131]
	Pseurotin A	Fungal metabolite	Tested in vitro and in vivo	[132]
	Silibinin A	TCM: polyphenol	Clinically used for treating liver diseases Tested in vitro +/− Statins	[133] [134]
	MK-0616	Tricyclic peptide	Tested in humans Phase I and II clinical trials	[135] [136]
Inhibiting PCSK9 expression	CRISPR/Cas9	Genome editing	Tested in vitro and in vivo in cynomolgus monkeys and macaques	[137,138]
	ISIS 394814/BMS 844421	ASO	Pre-clinically evaluated in vivo (terminated)	[139]
	SPC4061/SPC5001	ASO	Tested in vivo Clinically tested in humans but caused acute kidney injury (terminated)	[140,141] [142]
	Inclisiran (lequivo^®^)	siRNA	Approved in Europe Passed all three phase III ORION studies 160 Pending FDA approval	[143,144] [145] [146,147]
	R-IMPP	Pharmaceutical Molecule	Tested in vitro	[148]
	PF-06446846	Pharmaceutical Molecule	Tested in vivo	[149]
